# Report upon a Specimen of Xanthic Oxide Calculus

**Published:** 1883-02

**Authors:** W. W. Keen

**Affiliations:** Surgeon to St. Mary’s Hospital


### REPORT UPON A SPECIMEN OF XANTHIC OXIDE CALCULUS.

By W. W. KEEN, M. D., Surgeon to St. Mary's Hospital. [Read December 6, 1882.]

I desire to present for Dr. George L. Porter, of Bridgeport, Conn., a specimen of xanthic oxide calculus. It consists of one-half of a stone, an inch and a half long and an inch wide. This half is to be deposited in the Museum of the Jefferson Medical

College, the other half is in the Army Medical Museum, Washington, D.C.

So rare is this calculus that, including the present specimen, only eight have ever been described, and none of them so completely as Dr. Porter's. Moreover, this is the only specimen ever recognized and described by an American surgeon. Four of the specimens are British, two French, and one German. Marcet in 1877 described the first calculus of this kind which was recognized.

The present stone was passed spontaneously by a woman eighteen years old. Its clinical history presents nothing specially worthy of note, but its chemical constitution makes it very interesting. Xanthine or xanthic oxide is analogous to uric acid, having, however, one less equivalent of oxygen, and is the rarest of all calculi. In the *New England Medical Monthly* for May, 1882, Dr. Porter relates the case in full, with a drawing of the stone, and an analysis and comparison of the eight cases on record and some interesting remarks on xanthic oxide itself.

[After the reading of the preceding paper :—]

Dr. JOHN B. ROBERTS stated that in 1873, Dr. R. J. Levis operated for vesical calculus by lithotrity on a man. The patient, who was 69 years, was an inmate of the Pennsylvania Hospital. The fragments were examined by the late Dr. H. B. Hare, the well-known pathological chemist, and found to consist of xanthic oxide. The patient was discharged by request of his friends, while some of the stone still remained in the bladder, and passed from the surgeon's observation.

Dr. JAMES TYSON said that in connection with the case just reported by Dr. Keen, he desired to place on record a case which came under his own observation, of persistent cystin sediment in urine, concurrent with impacted oxalate of lime calculus. G. B. W., a very intelligent physician residing in one of the southern counties of Pennsylvania, and 45 years of age when he first saw him, was lithotomized in Baltimore when he was 28 years old, and a calculus of pure cystin removed. From that time he continued, according to his own account, to pass cystin daily. Early in January, 1879, a specimen of urine was sent to Dr. Tyson in which there was considerable pus and a proportionate amount of albumen. In this specimen there was found no cystin, but in later specimens there were found large numbers of the characteristic crystals along with pus and albumen. A little later Dr. Tyson visited him at his home, and found him suffering greatly with extreme lumbar pain, attacks like which he had frequently had before, but the present one was of unusual duration, and had greatly prostrated him. There seemed every reason to believe that there was a calculus impacted somewhere between the left kidney and the bladder. His sufferings continued, and he was only relieved by death, which occurred on the 6th of March, 1879.

The following notes of the autopsy were received from Dr. Wm. B. Rowland of Rowlandsville, Md.: The post-mortem examination revealed calculus in the left ureter just ready to pass into the bladder. The calculus was the size of a large pea, and very rough. Just behind where the calculus was found in the ureter was a collection of pus, dipping down into the pelvis, which would soon have made its exit through the ischiatic foramen if life had been prolonged.

The left kidney was somewhat enlarged, and the right was not more than one-fifth the usual size, but apparently healthy. The bladder, stomach, and bowels were healthy. No mention was made by Dr. Rowland of the liver, which was presumably healthy, but there was found in the gall-bladder a calculus an inch long and half an inch in diameter throughout its length.

The calculus which is exhibited to the College presented none of the physical and chemical characters of cystic calculi, which are smooth and friable, but is evidently oxalate of lime. This is particularly interesting in view of the fact that a cystin calculus was removed by lithotomy seventeen years earlier, and that the patient so persistently passed cystin crystals up to the time of his death.

